# Advances in Minimally Invasive Surgery for Biliary Tract Cancer from the Viewpoint of Liver Surgery: A Task-Based Framework for Disease-Specific Procedures

**DOI:** 10.3390/cancers18182957

**Published:** 2026-09-13

**Authors:** Zenichi Morise, Hiroyuki Kato, Akihiko Horiguchi, Hidetoshi Katsuno

**Affiliations:** Department of Gastroenterological Surgery, Fujita Health University School of Medicine, Bantane Hospital, 3-6-10 Otobashi, Nakagawa-ku, Nagoya 454-8509, Aichi, Japan

**Keywords:** minimally invasive surgery, biliary tract cancer, intrahepatic cholangiocarcinoma, perihilar cholangiocarcinoma, gallbladder cancer, distal cholangiocarcinoma, robotic surgery, laparoscopic surgery, lymphadenectomy, bile duct reconstruction

## Abstract

Biliary tract cancer (BTC) surgery often requires more than organ resection, and the role of minimally invasive surgery (MIS) cannot be adequately interpreted by disease subtype alone because operative complexity varies substantially within each subtype. This narrative review evaluates recent evidence for laparoscopic and robotic surgery in BTC from a task-based perspective, focusing on liver resection, lymphadenectomy, bile duct resection and reconstruction, vascular or multivisceral procedures, and pancreatic resection. Peripheral intrahepatic cholangiocarcinoma, which is often of the small-duct type, represents the most established field for minimally invasive liver resection, although lymphadenectomy remains inconsistent. Central intrahepatic cholangiocarcinoma, which is often of the large-duct type, should be considered separately because increasing hilar involvement may require systematic lymphadenectomy and bile duct resection and reconstruction, making the operation more similar to that for perihilar cholangiocarcinoma. Perihilar cholangiocarcinoma remains the highest-task-load frontier of BTC-MIS, typically requiring major liver resection with caudate lobectomy, bile duct resection, lymphadenectomy, and reconstruction of multiple small bile ducts. Current evidence is derived predominantly from retrospective studies of highly selected patients treated at expert centers. Gallbladder cancer should be stratified into Tis/T1a disease, T1b/T2 and selected T3 disease suitable for standard extended cholecystectomy, bile duct-involved disease, and locally advanced disease. Distal cholangiocarcinoma should be evaluated within the evidence base for minimally invasive pancreaticoduodenectomy rather than liver surgery. Overall, current evidence most strongly supports MIS for selected patients with peripheral intrahepatic cholangiocarcinoma and for standard extended cholecystectomy in selected patients with gallbladder cancer. Minimally invasive pancreaticoduodenectomy may also be considered for selected patients with distal cholangiocarcinoma in experienced centers. Central intrahepatic cholangiocarcinoma, perihilar cholangiocarcinoma, bile duct-involved gallbladder cancer, and locally advanced gallbladder cancer require cautious patient selection, standardized reporting, and prospective multicenter validation.

## Simple Summary

Biliary tract cancers include intrahepatic cholangiocarcinoma, perihilar cholangiocarcinoma, gallbladder cancer, and distal cholangiocarcinoma. Although laparoscopic and robotic surgery have expanded in hepatobiliary and pancreatic surgery, their role in biliary tract cancer remains difficult to define because the required procedures vary widely according to disease subtype and extent. Some tumors mainly require liver resection, whereas others require lymph node dissection, bile duct resection and reconstruction, vascular reconstruction, or pancreaticoduodenectomy. This review proposes a task-based framework for evaluating minimally invasive surgery according to the operative components required for oncologic resection. The strongest current evidence supports minimally invasive surgery for selected patients with peripheral intrahepatic cholangiocarcinoma and standard extended cholecystectomy in selected patients with gallbladder cancer. In contrast, central intrahepatic cholangiocarcinoma, perihilar cholangiocarcinoma, bile duct-involved gallbladder cancer, and locally advanced gallbladder cancer remain more complex surgical settings. Robotic surgery may facilitate precise dissection and reconstruction, but current evidence is mainly retrospective and based on highly selected patients.

## 1. Introduction

Biliary tract cancer (BTC), including intrahepatic cholangiocarcinoma (iCCA), perihilar cholangiocarcinoma (pCCA), gallbladder cancer (GBC), and distal cholangiocarcinoma (dCCA), remains a group of aggressive malignancies for which complete surgical resection is the only potentially curative treatment in selected patients [1,2]. Although minimally invasive surgery (MIS) has become widely accepted in many gastrointestinal and hepatobiliary procedures, its adoption for BTC has been more cautious because oncologic BTC surgery often requires a variable combination of liver resection (LR), regional lymphadenectomy, bile duct resection and reconstruction, and occasionally vascular or multivisceral resection [3,4]. Therefore, the role of MIS in BTC cannot be adequately assessed by disease name alone. It should instead be interpreted according to the operative task-load required to achieve oncologic quality.

From the viewpoint of liver surgery, the development of minimally invasive liver resection (MILR) has provided the technical foundation for applying MIS to selected BTC procedures. Improved parenchymal transection, better hemostatic strategies, the conceptual merit of the “caudal approach”, and consensus-based standardization have expanded the indications of MILR, including malignant liver tumors [5,6,7]. However, BTC surgery differs from LR for hepatocellular carcinoma or colorectal liver metastases because the oncologic operation frequently extends beyond hepatic parenchymal resection. In BTC, the adequacy of lymphadenectomy, the achievement of negative ductal and radial margins, the safety of biliary-enteric reconstruction, and, in selected cases, vascular resection and reconstruction directly influence both perioperative risk and oncologic validity [3,4].

This distinction is particularly important in iCCA. The oncologic surgery for peripheral or small-duct-type iCCA is usually dominated by LR, with lymphadenectomy performed selectively or routinely according to tumor location, nodal risk, and institutional policy. In contrast, central or large-duct-type iCCA frequently requires a higher task-load, including (often major) LR, more extensive lymphadenectomy, and, in advanced central tumors, extrahepatic bile duct resection and reconstruction. When tumor extension approaches the hepatic hilum, central iCCA may become surgically closer to pCCA than to conventional peripheral iCCA treated by LR alone [8,9,10].

pCCA represents the highest task-load category among BTC procedures. Curative-intent treatment typically requires major LR with caudate lobectomy, en bloc extrahepatic bile duct resection, multi-duct biliary reconstruction, and regional lymphadenectomy, with vascular resection and reconstruction in selected patients [11,12,13]. Recent laparoscopic and robotic series, including multicenter propensity score-matched analyses, suggest technical feasibility in selected cases [14,15,16,17]. Nevertheless, the evidence remains retrospective, heterogeneous, and strongly influenced by patient selection, institutional expertise, and learning-curve effects.

GBC also requires task-based stratification. Tis and T1a diseases are generally treated by simple cholecystectomy and should not be pooled with extended oncologic resections [18]. The principal comparative field for oncologic MIS in BTC comprises resectable T1b/T2 disease suitable for standard extended cholecystectomy, which generally consists of cholecystectomy with gallbladder bed resection or segment IVb/V LR plus regional lymphadenectomy [19,20,21]. Selected T3 tumors with only limited gallbladder bed invasion may also be amenable to this procedure. Tumors with cystic duct margin involvement or direct bile duct invasion may require bile duct resection and biliary reconstruction. Those and the other locally advanced GBCs, requiring major LR or vascular/multivisceral resection, should be evaluated separately [22].

dCCA requires a different interpretive framework. Although dCCA shares the tasks of bile duct resection and regional lymphadenectomy with pCCA and selected GBC, its standard curative procedure is pancreaticoduodenectomy (PD) rather than LR. Therefore, the relevant evidence base is minimally invasive pancreaticoduodenectomy (MIPD), including laparoscopic and robotic PD [23,24,25,26].

The purpose of this narrative review is to propose a task-based framework for interpreting the evolution and future perspectives of MIS in BTC from the viewpoint of liver surgery. This framework clarifies why MIS is most mature for selected peripheral iCCA and standard GBC extended cholecystectomy, why pCCA and central iCCA requiring biliary reconstruction remain high-complexity frontiers, and why dCCA should be discussed within the MIPD evidence base. Furthermore, this review uses the proposed framework to examine whether robotic assistance can facilitate the operative components that have historically constrained conventional laparoscopic surgery for BTC.

In this narrative review, disease- and procedure-specific evidence on MIS for BTC was gathered and evaluated. Relevant English-language systematic reviews, meta-analyses, comparative cohort studies, propensity score–matched analyses, multicenter series, and landmark feasibility or technical reports published from 2005 onward were identified through database searches of MEDLINE, Embase, and the Cochrane Library, supplemented by screening of key reference lists. The final literature search was performed on 10 August 2026. Search terms included combinations of disease-specific terms (“biliary tract cancer,” “cholangiocarcinoma,” “intrahepatic cholangiocarcinoma,” “perihilar cholangiocarcinoma,” “gallbladder cancer,” and “distal cholangiocarcinoma”) and procedure- or approach-specific terms (“minimally invasive surgery,” “laparoscopic surgery,” “robotic surgery,” “liver resection,” “hepatectomy,” “lymphadenectomy,” “bile duct resection,” “biliary reconstruction,” and “pancreaticoduodenectomy”), combined using “AND” and “OR.” The evidence was narratively summarized by BTC subtype because of heterogeneity in study design, patient selection, and operative procedures.

## 2. Intrahepatic Cholangiocarcinoma: Peripheral LR-Dominant Disease and Central High Task-Load Disease

iCCA is the second most common primary liver malignancy after hepatocellular carcinoma and remains a disease for which only complete surgical resection in selected patients offers the chance of cure or long-term survival [27,28,29]. However, iCCA is not a single operative entity from the viewpoint of oncological surgical tasks. The tasks for peripheral or small-duct-type iCCA, often mass-forming and associated with chronic liver diseases like hepatocellular carcinoma, are usually dominated by tumor-oriented LR, with lymphadenectomy performed according to nodal risk and institutional strategy [27,29,30,31,32]. By contrast, central or large-duct-type iCCA, which has more invasive features, may require major LR, regional lymphadenectomy, and, in advanced central tumors, extrahepatic bile duct resection with reconstruction. When tumor extension approaches the hepatic hilum, central iCCA may become surgically closer to pCCA than to peripheral iCCA treated mainly by LR [8,9].

Lymphadenectomy is one of the most important unresolved issues in iCCA surgery. Nodal status is a major prognostic factor, and adequate nodal evaluation is necessary for accurate staging and postoperative treatment planning. AJCC-oriented recommendations and expert discussions commonly refer to evaluation of at least six lymph nodes as a practical benchmark for adequate staging [31,32]. However, real-world adherence remains low, and MIS series frequently report inconsistent lymphadenectomy rates and low nodal yields [3,33].

Umeda et al. analyzed 279 patients who underwent lymphadenectomy and classified nodal basins as Level 1, hepatoduodenal ligament nodes; Level 2, postpancreatic or common hepatic artery nodes; and Level 3, gastrocardiac, left gastric artery, or celiac artery nodes. Lymph node metastasis was present in 39% of patients, and 5-year survival decreased according to nodal extent: 45.3% for N0 disease, 27.1% for Level 1 metastasis, 22.9% for Level 2 metastasis, and 7.3% for Level 3 metastasis [8]. Tumor location-based analyses suggest that central iCCA may justify lymphadenectomy beyond the hepatoduodenal ligament, whereas lymphadenectomy for peripheral iCCA has much less therapeutic benefit, mostly deriving only nodal-staging value [8,9].

Systematic reviews and propensity score-matched analyses generally report perioperative advantages for laparoscopic compared with open LR, including reduced blood loss, fewer major complications, and shorter hospital stay, while showing broadly comparable long-term survival in selected cohorts of iCCA [33,34,35,36,37]. The most recent comparative meta-analysis included 37 non-randomized studies and 4863 patients with intrahepatic or perihilar cholangiocarcinoma, including 21 propensity score-matched studies. In the iCCA subgroup, MILS was associated with lower major morbidity, reduced blood loss, and shorter hospital stay than open surgery. However, tumors selected for MILS were smaller, and substantial heterogeneity remained in operative extent, lymphadenectomy, and patient selection [37]. These findings support selective MIS for peripheral iCCA when LR is the dominant task. Nevertheless, the available studies generally did not distinguish peripheral/small-duct-type from central/large-duct-type iCCA, and patients selected for MIS often had more favorable tumor location, smaller tumor burden, less need for major LR, and a lower likelihood of extensive lymphadenectomy or bile duct resection [33,34,35,36,37]. Therefore, the pooled evidence should not be extrapolated directly to central iCCA requiring high-task-load hilar surgery. iCCA is currently the most mature BTC subtype for MILR, but it also demonstrates that competence in LR alone does not necessarily ensure oncologic completeness in BTC surgery [3,33,34,35,36,37].

Robotic assistance may theoretically address some technical limitations of conventional laparoscopy in iCCA, particularly when the operation requires meticulous hilar dissection, lymphadenectomy, or deep dissection around major vascular structures [4,38]. Nevertheless, current evidence does not yet demonstrate that robotic surgery improves oncologic outcomes in iCCA compared with conventional laparoscopic or open surgery. Quach and colleagues frame this as part of a broader hepatobiliary evolution: as robotic platforms integrate into structured programs, complex oncologic operations may become more reproducible without sacrificing oncologic quality, though they stress the need for training centrality, procedural standardization, and appropriate case selection [4,38,39]. At the moment, the potential advantages of robotics should be regarded as technical hypotheses or aids to task completion rather than established clinical benefits.

In future studies of MIS for iCCA, the biological characteristics, operative requirements, and treatment strategies of peripheral and central iCCA should be more clearly distinguished. The BTC-focused update reported by Itano et al. captures the present reality: while the number of MIS iCCA reports is increasing and feasibility is supported in expert hands, oncologic equivalence and generalizability remain unsettled, largely because studies are retrospective, heterogeneous, and highly center-dependent [3]. Besides the examination of long-term outcomes, prospective comparative studies and/or international registries with standardized reporting of iCCA-MIS should be expected. Such studies should report on the need for major liver resection, bile duct resection and reconstruction, and lymphadenectomy as baseline operative characteristics. Thereafter, they should also assess nodal yield, parenchymal and ductal margin status, conversion and reasons for conversion, postoperative morbidity, recurrence patterns, disease-free survival, and overall survival as outcomes. With these clarifications, universal indications and advantages/disadvantages of iCCA-MIS (laparoscopic and/or robotic procedures) can be discussed.

## 3. Perihilar Cholangiocarcinoma: The Highest Task-Load Frontier of BTC-MIS

pCCA represents the highest task-load category among BTC procedures. Curative-intent resection usually requires simultaneous completion of major liver resection with caudate lobectomy, en bloc extrahepatic bile duct resection, (often multiple small) biliary-enteric reconstructions, and regional lymphadenectomy. Furthermore, portal vein (and/or hepatic artery) resection and reconstruction may also be applied to achieve R0 resection in selected patients [11,12,13].

The open approach remains the reference standard for most pCCA resections because incomplete execution of any component of the operation may compromise oncologic quality. R0 resection depends not only on liver transection but also on proximal and distal bile duct margins, the radial hilar dissection margin, and clearance of vascular involvement. Thus, the central question for MIS in pCCA is whether the entire pCCA task stack can be reproduced with the same oncologic fidelity and safety as open surgery [3,4].

Early MIS experiences, predominantly laparoscopic surgery, in pCCA were largely limited to case reports and small series [40,41,42,43,44,45]. Persistent skepticism was driven by concerns regarding oncologic completeness, particularly inconsistent caudate lobectomy, variable lymphadenectomy, and the technical difficulty of multi-duct biliary reconstruction using conventional straight laparoscopic instruments [40,42]. Earlier systematic assessments emphasized that MIS for pCCA may be feasible in highly specialized high-volume centers, but cautioned that omission of oncologically important components could compromise staging accuracy, local control, and oncologic quality [40]. More recent comparative studies have reported encouraging short-term outcomes in selected patients treated at high-volume centers. Wang et al. compared 256 laparoscopic and 389 open pCCA resections in a multicenter propensity score-matched study [14]. Qin et al. reported comparable perioperative and survival outcomes between laparoscopic and open approaches after multicenter propensity score matching [15]. Systematic reviews have concluded that MIS for pCCA may achieve acceptable short-term outcomes in highly specialized centers, but long-term oncologic data remain insufficient to establish generalizable equivalence [16,37,42]. In the pCCA subgroup (from seven studies) of the most recent comparative meta-analysis, major morbidity and mortality were not significantly different between minimally invasive and open surgery. MIS was associated with an approximately 85 min longer operation time and a 1.5-day shorter hospital stay, whereas blood loss did not differ significantly [37]. Bile leakage was also comparable in the subgroup undergoing biliodigestive reconstruction. Nevertheless, all included evidence was non-randomized, and robotic cases remained limited.

Robotic surgery may be attractive for pCCA because three-dimensional visualization, articulated instruments, tremor filtration, and improved ergonomics may facilitate fine hilar dissection, lymphadenectomy, suturing of multiple small bile ducts, and selected vascular reconstruction [4,38]. Initial reports support technical feasibility of robotic pCCA surgery [43,44]. However, these experiences remain small and concentrated in specialized centers [16,42]. An early propensity score-matched study by Efanov et al. addressed robotic versus open pCCA surgery in a single-center experience. Forty-one robotic cases were matched with 82 open cases; 90-day mortality was 5% in both groups, and median overall survival was 44 months in the robotic group and 29 months in the open group without a significant difference [17]. Although this study begins to discuss a potential safe boundary for robotic pCCA surgery, it is a single-center retrospective study with strict patient selection in a highly specialized center. Parallel systematic reviews of minimally invasive and robotic approaches for pCCA have supported technical feasibility and acceptable short-term perioperative outcomes in carefully selected small cohorts, while consistently emphasizing that the available evidence remains limited, heterogeneous, and insufficient to establish definitive long-term oncologic equivalence or justify widespread adoption beyond expert centers [3,40,42]. Variability in key operative components—including caudate lobectomy, bile duct margin management, lymphadenectomy, and reconstruction techniques—continues to limit comparability across studies. Furthermore, even if robotics provides advantages for lymphadenectomy and for the dissection, suturing, and reconstruction of bile ducts and vessels, they, in their current bulky and space-requiring form, are not so advantageous for extended major LR handling a large and space-occupying liver inside the limited space in the subphrenic “rib cage” [39].

Because pCCA surgery combines multiple high-risk oncologic tasks, patient selection is one of the central points in comparative studies. Factors that should be reported and examined include Bismuth–Corlette classification, longitudinal and radial tumor extent, number and size of expected biliary-enteric anastomoses, future liver remnant volume and function, need for portal vein embolization, vascular involvement, caudate lobe invasion, prior biliary drainage, cholangitis, and institutional experience. For pCCA-MIS to move beyond feasibility, future studies should also evaluate standardized quality metrics including 1. operative metrics: R0 resection including proximal ductal and radial margins, completion of caudate lobectomy, lymph node yield and nodal stations, and conversion and reasons for conversion; 2. postoperative short-term metrics: bile leakage, post-LR liver failure, major morbidity, re-hospitalization, 90-day mortality, and time to adjuvant therapy; and 3. postoperative long-term oncologic metrics: disease-free survival, recurrence pattern, and overall survival. Large-scale multicenter prospective studies and registry-based analyses with the evaluation of these factors and/or a randomized controlled trial could show the real possibility of generalization, indications, and advantages/disadvantages of the MIS procedure for pCCA and may lead to further expansion of the role of MIS in pCCA. At present, the importance of centralization, structured learning-curve assessment, and centralized prospective studies are emphasized [3,16,40,42].

## 4. Gallbladder Cancer: Stage- and Extension-Based Task Stratification

GBC represents a heterogeneous field for MIS because operative task-load varies markedly according to T stage, cystic duct or bile duct involvement, liver or hilar invasion, nodal disease status, and vascular or adjacent organ involvement. At least four scenarios should be separated: Tis/T1a disease treated by simple cholecystectomy; T1b/T2 and selected T3 disease suitable for standard extended cholecystectomy with LR and lymphadenectomy; tumors with cystic duct margin involvement or extrahepatic bile duct invasion requiring bile duct resection and biliary reconstruction; and locally advanced disease requiring major LR (sometimes with multiple small bile duct reconstructions), vascular resection or multivisceral resection [18,19,20,21,22]. Selected T3 tumors with only limited gallbladder bed invasion may be included in the second category and should be distinguished from locally advanced tumors.

Tis and T1a GBC constitute the lowest task-load category. Simple cholecystectomy is generally considered sufficient for Tis/T1a disease when complete excision is achieved [18]. These cases should be separated from studies evaluating oncologic extended cholecystectomy.

The principal field for evaluating MIS in GBC is T1b/T2 disease suitable for standard extended cholecystectomy. Selected T3 tumors with only limited gallbladder bed invasion may also be amenable to this procedure, without bile duct or cystic duct involvement, hilar or vascular invasion, adjacent-organ invasion, or liver metastases. Standard oncologic surgery in this setting generally consists of cholecystectomy with gallbladder bed resection or segment IVb/V LR and regional lymphadenectomy (mainly around hepatoduodenal ligament). Once surgery requires liver bed LR and regional lymphadenectomy, the procedure is no longer a simple cholecystectomy nor LR but rather a complex oncologic resection in which the technical quality of each task may directly influence recurrence risk and oncologic outcomes [19,21,46]. There has been discussion about the need for bile duct resection in those cases. In a Japanese Biliary Tract Cancer Registry study of 1609 patients with pT2 GBC without distant metastasis, overall disease-specific survival did not differ between patients with and without extrahepatic bile duct resection, 76% versus 79%, whereas Clavien–Dindo grade 3 or higher complications were higher with bile duct resection, 32.4% versus 11.7% [22]. These findings do not support routine extrahepatic bile duct resection in the absence of direct bile duct involvement or a positive cystic duct margin. Without the need for bile duct resection, this category represents an intermediate task-load procedure less reconstructive than pCCA.

Recent comparative studies and meta-analyses suggest that MIS may be feasible for standard extended cholecystectomy in selected patients and experienced centers. A network meta-analysis including 32 studies and 5883 patients reported that MIS was associated with shorter hospitalization, less blood loss, and fewer complications than open surgery, while overall survival, R0 resection, and lymph node harvest were not significantly different among approaches [19]. The IRON study, an international multicenter retrospective cohort study comparing robotic, laparoscopic, and open approaches for radical resection of T1b-T3 GBC, showed lower blood loss and higher lymph node yield with robotic surgery after propensity score matching, while overall and disease-free survival were similar across approaches [20]. Another multicenter propensity score-matched study of minimally invasive extended cholecystectomy without bile duct invasion also reported comparable R0 resection, lymph node yield, morbidity, and survival compared with open surgery [21]. A 2026 meta-analysis restricted to five propensity score-matched studies, including 629 patients, further showed that robotic radical cholecystectomy was associated with fewer major complications, lower blood loss, and a shorter hospital stay than open surgery [47]. However, lymph node yield, R0 resection, mortality, and 3- and 5-year overall and disease-free survival did not differ significantly. Thus, current pooled evidence supports potential perioperative benefits of robotic surgery without demonstrating oncologic superiority [47]. The available literature remains heterogeneous with respect to tumor stage, extent of lymphadenectomy, and type of liver resection [3,46,48]. Laparoscopic GBC surgery has matured primarily in settings in which higher-level components of the BTC task stack, such as extensive lymphadenectomy, bile duct resection, and complex reconstruction, are absent or limited [48]. Extension of MIS indications to advanced GBC requiring radical lymphadenectomy, major LR, or bile duct resection and reconstruction therefore remains controversial [3,19,46,47,48].

GBC with cystic duct margin involvement or direct extrahepatic bile duct invasion should be considered a separate high-task-load subgroup. In these patients, extrahepatic bile duct resection and biliary-enteric reconstruction are necessary to achieve a negative margin. In the aforementioned Japanese Biliary Tract Cancer Registry study, bile duct resection was also associated with improved 5-year survival, 64% versus 54%, in selected T2N1 patients undergoing gallbladder bed LR [22]. However, this retrospective subgroup finding does not establish lymph-node metastasis alone as an indication for bile duct resection.

Recent robotic series have reported the feasibility of bile duct resection and reconstruction in selected GBC patients [48]. Among patients undergoing fully robotic liver resection for GBC, an international multicenter propensity score-matched comparison of procedures with versus without common bile duct resection found no significant differences after matching in blood loss, conversion, bile leak, major complications, length of stay, lymph node yield, recurrence, 90-day mortality, overall survival, or disease-free survival [49].

Robotics may change the role of MIS in GBC by improving the reproducibility and precision of procedures performed within hepatoduodenal ligament and hilar spaces [48,50]. Reviews focusing on robotic innovations in GBC surgery highlight the advantages of enhanced three-dimensional visualization and articulated instrumentation for lymphadenectomy and dissection of vessels and bile duct around the hepatoduodenal ligament [48,50]. These technical characteristics may facilitate more consistent nodal dissection and reduce operative morbidity in experienced hands, particularly when the oncologic quality of surgery depends more on those procedures than on organ (gallbladder and liver bed) resection alone [38,48,50].

Nevertheless, robust comparative evidence remains limited, and current data are derived predominantly from retrospective series and highly selected institutional experiences [3]. Importantly, the field has begun to move toward prospective evaluation. The ROBOCOP trial, an ongoing randomized study comparing robotic and open resection for GBC, could be an important transition from demonstrating technical feasibility toward defining appropriate indications, oncologic value, and the potential role of robotics in standard GBC care [3,51].

Locally advanced GBC should be evaluated separately from disease suitable for standard extended cholecystectomy. As GBC progresses, the LR component may expand from gallbladder bed resection or segment IVb/V LR to major LR, often with bile duct resection and reconstruction. Lymphadenectomy may become more extensive, and vascular resection or multivisceral resection may be required. In tumors extending toward the hepatic hilum, the anatomy and task-load may become similar to pCCA. For locally advanced GBC, evidence supporting MIS remains limited and it should be regarded as exceptional or investigational rather than routine.

## 5. Distal Cholangiocarcinoma: Bile Duct Resection and Lymphadenectomy Within the Pancreaticoduodenectomy Framework

dCCA differs fundamentally from the other BTC subtypes because its standard curative procedure is pancreaticoduodenectomy (PD), not associated with LR. From a task-based perspective, dCCA shares several oncologic tasks with pCCA and selected GBC, particularly bile duct resection with ductal margin control and reconstruction, and regional lymphadenectomy around the hepatoduodenal ligament, common hepatic artery, and celiac artery. However, the dominant operative component is pancreatic resection with pancreatic–enteric reconstruction and, in contrast to pCCA, dCCA does not usually require LR or multi-duct hilar reconstruction. Therefore, the role of MIS in dCCA should be evaluated within the evidence base of MIPD rather than MILR [23,24,25,26,52,53,54].

Open PD remains the reference standard for resectable dCCA. Oncologic quality depends on R0 resection, adequate regional lymphadenectomy, appropriate bile duct and pancreatic margins, and safe reconstruction. Current interest centers on whether minimally invasive PD (MIPD) can reproduce the oncologic quality of open surgery while facilitating postoperative recovery, which may support timely initiation of adjuvant therapy [3,23,53]. As in other BTC procedures, oncologic validity depends on successful completion of R0 resection, which includes lymphadenectomy, deep dissection, and complex reconstruction besides resection itself [3,4]. The adoption of laparoscopic PD has been slow because of the technical demands of resection, lymphadenectomy, and multiple anastomoses [3].

In an international propensity score-matched cohort study, Uijterwijk et al. compared 97 MIPD cases, including 37 robotic and 60 laparoscopic PDs, with 381 open cases. MIPD was associated with less blood loss, 300 versus 420 mL, and fewer surgical site infections, but longer operation time, 453 versus 336 min. Major complications were similar, 23% versus 20%, and lymph node retrieval was comparable, 14 versus 14 nodes. Five-year overall survival and disease-free survival were also comparable, 35% versus 24% and 25% versus 21%, respectively [23]. Additional comparative studies and meta-analyses support the same general interpretation [24,25,26]. Gao et al. evaluated laparoscopic versus open PD after completion of the learning curve in 430 patients, with 184 patients in each group after propensity score matching. Laparoscopic PD was associated with less blood loss, 160 versus 250 mL, and shorter postoperative length of stay, 13 versus 16 days, while operation time, lymph node harvest, R0 resection, morbidity, readmission, 30-day mortality, and long-term prognosis did not differ significantly [25]. Systematic reviews and meta-analyses generally support the feasibility of MIPD for selected dCCA patients. Domene et al. analyzed 1349 patients and found no significant differences in transfusion, complications, or mortality [53]. Bakht et al. included seven retrospective cohort studies for their meta-analysis comprising 1803 patients and found that MIPD reduced blood loss and hospital stay but increased operation time; major complications, mortality, overall survival, and disease-free survival were not significantly different [26].

Robotic PD may overcome some limitations of conventional laparoscopic PD by improving suturing, reconstruction, and deep dissection around the pancreatic head and bile duct. Xu et al. compared robotic and open PD for dCCA after propensity score matching and reported lower estimated blood loss, 150 versus 250 mL, and shorter postoperative length of stay, 12 versus 15 days, with no significant differences in lymph node harvest, R0 resection, major morbidity, 90-day mortality, or long-term survival [24]. A multicenter propensity score-matched study found comparable oncologic and perioperative outcomes between robotic and laparoscopic PD for dCCA after completion of learning curves. The shorter operation time in the robotic group suggested a possible efficiency signal, although this finding may reflect surgeon experience, institutional workflow, case selection, and center volume [54]. Also in dCCA, the available evidence remains largely retrospective and prone to selection bias [53,55]. Therefore, oncologic equivalence to open PD remains unproven, and standardized reporting is required [3,23,53].

## 6. Discussion (Table 1)

**Figure 1:**
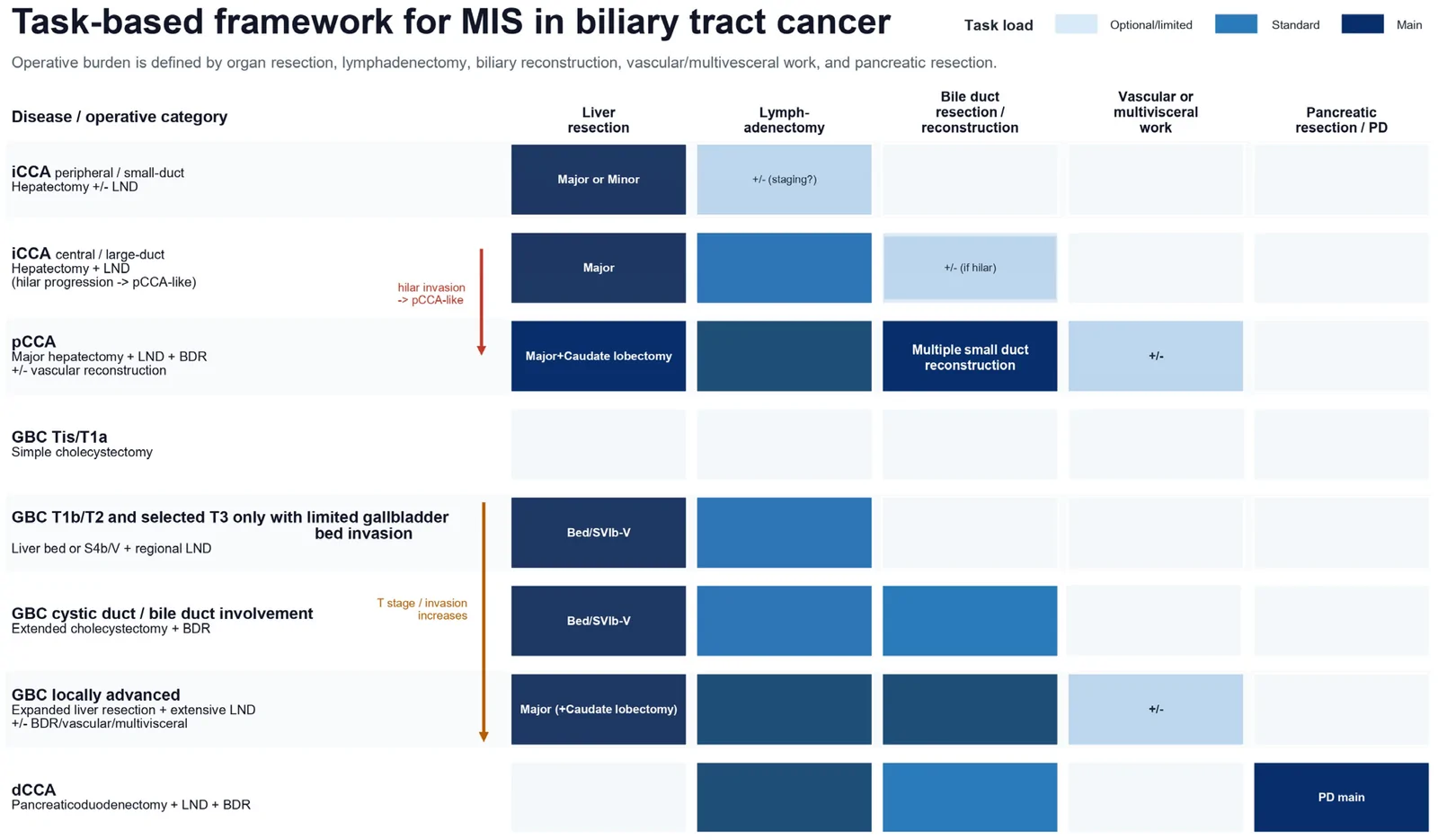
Task-based framework for minimally invasive surgery in biliary tract cancer. The framework depicts the relative contribution of five operative components: liver resection, lymphadenectomy, bile duct resection and reconstruction, vascular or multivisceral procedures, and pancreatic resection. Peripheral or small-duct-type iCCA is predominantly liver resection-oriented, whereas central or large-duct-type iCCA adds a greater lymphadenectomy burden and may require bile duct resection and reconstruction as tumor extension approaches the hepatic hilum, resulting in a pCCA-like operative profile [8,9,27,29]. pCCA represents the highest combined task-load category, typically requiring major liver resection with caudate lobectomy, regional lymphadenectomy, en bloc extrahepatic bile duct resection, multi-duct biliary reconstruction, and, in selected patients, vascular resection and reconstruction [11,12,13,14,15,16,17,37,40,42,43,44]. For GBC, Tis/T1a disease is generally treated by simple cholecystectomy, whereas resectable T1b/T2 and selected T3 disease is principally managed by extended cholecystectomy with gallbladder bed or segment IVb/V liver resection and regional lymphadenectomy [18,19,20,21,46,47,48]. Cystic duct margin involvement or direct extrahepatic bile duct invasion adds bile duct resection and biliary reconstruction, while hilar, vascular, multivisceral, or extensive hepatic involvement further increase the task-load and may create a pCCA-like operative profile [22,49,57]. dCCA overlaps with pCCA and selected GBC in bile duct resection, margin control, biliary reconstruction, and regional lymphadenectomy; however, pancreaticoduodenectomy, rather than liver resection, is the dominant organ-resection task [23,24,25,26,52,53,54,55]. Figure 1 Footnote. Color intensity represents the conceptual relative task-load of each operative component, ranging from absent or minimal to dominant or defining. The gradation is a qualitative synthesis of the operative requirements and available literature, not a validated numerical score or an indication algorithm. The figure is intended to support interpretation of disease- and procedure-specific MIS evidence. Individual treatment decisions must also account for tumor biology, anatomical extent, future liver remnant, patient fitness, institutional volume, surgeon experience, and learning-curve stage. Figure 1 Abbreviations. BTC, biliary tract cancer; MIS, minimally invasive surgery; iCCA, intrahepatic cholangiocarcinoma; pCCA, perihilar cholangiocarcinoma; GBC, gallbladder cancer; dCCA, distal cholangiocarcinoma; LND, lymph node dissection; BDR, bile duct resection and reconstruction; PD, pancreaticoduodenectomy; S4b/V, liver segments IVb and V.

The evolution of MIS for BTC is best understood as an escalation in the ability to reproduce a stacked set of oncologic tasks: organ resection (resections of liver with deep hilar exposure and stable hemostasis or pancreas with pancreatic–enteric reconstruction), adequate regional lymphadenectomy, high-fidelity biliary reconstruction (sometimes involving multiple small ducts), and, in selected cases, vascular resection and reconstruction (Figure 1).

Laparoscopic liver surgery matured through decades of technical and technological refinement, the development of conceptual frameworks, such as the “caudal approach”, and consensus-driven standardization [5,6,7,56]. However, BTC surgery is characterized by the unique combination of LR, lymphadenectomy, and bile duct resection/reconstruction, which helps explain both the delayed adoption of MIS and the higher threshold required to establish oncologic legitimacy compared with other hepatobiliary procedures [3,6,40,42].

With this background, the BTC-focused update and the broader hepatobiliary review represent two complementary perspectives that must be considered simultaneously [3,4]. While evidence supporting technical feasibility and short-term safety continues to accumulate, the oncologic impact of MIS remains uncertain. Long-term equivalence to open surgery is still debated across BTC subtypes, because available evidence is dominated by retrospective studies in specialized centers, and substantial heterogeneity in operative extent exists [3]. On the other hand, the broader evolution of minimally invasive hepatobiliary surgery demonstrates a progressive movement toward oncologic equivalence with improved perioperative outcomes. Robotic surgery is increasingly viewed as a platform that may facilitate this transition [4]. Ongoing technological innovation and structured programmatic integration may allow MIS to become more widely adopted without compromising oncologic standards [4]. Together, these perspectives define the future agenda for BTC-MIS: converting expanding technical capability into auditable and reproducible oncologic quality rather than relying on feasibility alone [3,4].

Disease-specific evidence supports this stratified interpretation. For peripheral iCCA, liver resection-dominant procedures represent the most mature BTC-MIS domain. Multiple systematic reviews and comparative analyses have demonstrated lower blood loss, fewer major complications, and shorter hospital stay, with broadly comparable long-term oncologic outcomes in selected patients [33,34,35,36,37]. However, these studies generally did not distinguish peripheral/small-duct-type iCCA from central/large-duct-type iCCA, and the available evidence remains affected by tumor-size imbalance, inconsistent lymphadenectomy, and selection of lower task-load procedures for MIS [37]. A persistent concern is the low and variable performance of lymphadenectomy in MIS series despite its prognostic and staging value [33]. Future oncologic assessments should therefore separate peripheral and central iCCA, and report the extent of lymphadenectomy, bile duct resection, reconstruction, and major LR based on the oncologic need of each task.

For GBC, the field has progressed from a historical contraindication for laparoscopy, largely related to concerns regarding gallbladder perforation, bile spillage, and tumor dissemination, toward selective laparoscopic and robotic extended oncologic resection [19,46,48,57]. Nevertheless, adequacy of lymphadenectomy and, in advanced patients, the need for major LR and biliary resection and reconstruction continue to limit broader acceptance [19,46,48]. GBC should therefore be stratified according to the operative task-load required by each oncologic condition. A recent meta-analysis restricted to propensity score-matched studies further supports this interpretation: robotic radical cholecystectomy was associated with fewer major complications, lower blood loss, and shorter hospitalization, but no significant differences were demonstrated in R0 resection, lymph node yield, or 3- and 5-year overall and disease-free survival [47]. Accordingly, robotic surgery may offer perioperative advantages, but current evidence does not demonstrate oncologic equivalence or superiority. The ROBOCOP randomized study reflects the growing recognition that high-level prospective evidence is required to define the oncologic role of robotic surgery in GBC [3,51].

Perihilar cholangiocarcinoma remains the ultimate test for BTC-MIS because it requires simultaneous completion of major LR with caudate lobectomy, en bloc bile duct resection, meticulous regional lymphadenectomy, and multi-duct biliary reconstruction, with vascular resection and reconstruction in selected patients [40,42,44]. These components magnify both technical complexity and the oncologic consequences of incomplete task execution [40,42]. Robotics may represent a qualitative change rather than a simple extension of laparoscopy because articulated instruments can facilitate multi-duct biliary reconstruction and fine hilar dissection [43,44]. The most recent comparative meta-analysis showed that, in the pCCA subgroup from seven studies, MIS was associated with a longer operation time and shorter hospital stay, without significant differences in major morbidity or mortality. In a separate subgroup undergoing biliodigestive anastomosis, bile leakage was comparable between MIS and open surgery [37]. These pooled findings support feasibility but remain insufficient to establish equivalence because the evidence was entirely non-randomized and robotic cases were few. Contemporary reviews also emphasize heterogeneity in lymphadenectomy, caudate resection, biliary reconstruction, and vascular procedures [3,40,42]. Wider adoption of robotic surgery requires substantial capital investment, maintenance resources, disposable instruments, operating-room coordination, and platform-specific training. Furthermore, current robotic systems remain relatively bulky and may offer less advantage during mobilization and handling of a large, space-occupying liver inside the limited space of the subphrenic “rib cage” [39]. Learning-curve effects, institutional experience, and procedural standardization should therefore be explicitly evaluated in future studies [3,37,42].

For dCCA, where the principal operative tasks are those of PD rather than LR, disease-specific analyses of MIPD suggest reduced blood loss and broadly comparable perioperative and oncologic outcomes relative to open surgery [23,53,55]. However, the available evidence is derived predominantly from retrospective studies, and systematic reviews consistently caution against assuming oncologic equivalence in the absence of more rigorous comparative data [3,53,55]. Although current evidence supports feasibility and selective use in experienced centers, broader generalization remains dependent on institutional expertise and standardization [3,53,54].

Currently, BTC-MIS has progressed beyond simple feasibility and is approaching a systematic redefinition of which oncologic tasks can be performed in a standardized minimally invasive manner, particularly lymphadenectomy and biliary reconstruction, which have traditionally distinguished BTC surgery from LR in MIS introduction [4,6,43]. Contemporary reviews consistently emphasize the importance of centralization of complex BTC surgery, competency-based training with defined learning curves, and prospective data collection using standardized outcome measures [3,4,51]. Endpoints for outcome measurement should include R0 status, lymph-node yield and nodal stations, conversion rate and reasons for conversion, postoperative morbidity and mortality, time to adjuvant therapy, and long-term oncologic outcomes [3,4]. When combined with these quality-control frameworks, task-based assessment may allow for MIS, including robotic surgery, to be evaluated as a means of reducing procedural variability and improving oncologic fidelity, ultimately determining whether MIS can be adopted more broadly without compromising oncologic quality [3,4,6].

**Table 1:** Task-Based operative categories and streamlined comparative evidence for minimally invasive surgery in biliary tract cancer. Table 1. Legend. This table prioritizes comparative studies, propensity score-matched analyses, and meta-analyses according to task-defined operative categories. Non-comparative studies are retained only when they define the reference operation or identify a major evidence gap. Perioperative advantages of MIS should be interpreted in light of patient selection, tumor burden, operative extent, lymphadenectomy quality, institutional volume, and learning-curve effects. Central/large-duct-type iCCA remains without a dedicated comparative MIS evidence base.

Disease/Operative Category	Standard Open Oncologic Procedure	Study: Author, Year, Study Type	Sample Size, Design	Short-Term Outcomes	Long-Term/Oncologic Outcomes
iCCA, peripheral/small-duct-type	Liver resection ± regional LND	de Hondt et al., 2025. [37] Systematic review/meta-analysis of 37 comparative non-randomized studies, including 21 PSM studies.	4863 patients overall (pCCA 1185, iCCA 3678) 2181 MILS vs 2662 open procedures.	In whole cohort including pCCA: Conversion was reported in 30 studies: 11.1% (range 0–40%). Overall R0: 90.4% vs. 81.4% (OR 1.40); LN yield: median 8 vs. 7, not significantly different. In the iCCA subgroup, major morbidity was 11.5% vs. 15.1% (OR 0.65); mortality was 2.3% vs. 3.5% (OR 0.65); hospital stay was 1.9 days shorter; and blood loss was 123 mL lower after MILS.	Recurrence 62.8% vs. 63.1%, not significantly different. (These were whole-cohort results including pCCA.)
		Aliseda et al., 2023. [34] Systematic review/meta-analysis of PSM studies.	1042 patients LLR vs. OLR.	Reduced bleeding by about 161 mL, lower transfusion, lower major complications (OR 0.60), and shorter stay by about 3.2 days.	Survival broadly comparable between LLR and OLR.
		Sahakyan et al., 2023. [36] Multicenter PSM.	50 LLR vs. 86 OLR.	Operation time 209 vs. 294 min; major complications 24% vs. 38%; R0 84% vs. 84%; median retrieved nodes 1 vs. 2.	5-year OS 47% vs. 43%; 3-year RFS 40% vs. 38%.
iCCA, central/large-duct-type	Extended liver resection ± extrahepatic bile duct resection and reconstruction + systematic LND	No comparative MIS study specifically limited to central/large-duct-type iCCA was identified.	Location-based oncologic evidence is provided by Umeda et al. and Endo et al. [8,9].	Not available for a central iCCA-specific MIS vs. open comparison.	Not available for a central iCCA-specific MIS vs. open comparison.
pCCA	Major liver resection + caudate lobectomy + bile duct resection/reconstruction + regional LND ± vascular work	Wang et al., 2023. [14] Multicenter PSM.	256 laparoscopic vs. 389 open.	Operation time 359 vs. 342 min; blood loss 300 vs. 300 mL; C-D ≥ III 12% vs. 23%; R0 82% vs. 84%; nodes 12 vs. 11.	Long-term outcomes were not the primary endpoint.
		Qin et al., 2023. [15] Multicenter PSM.	161 laparoscopic vs. 306 open.	Operation time 360 vs. 356 min; blood loss 300 vs. 300 mL; C-D ≥ III 12% vs. 19%; R0 94% vs. 88%; nodes 8 vs. 8.	Median OS comparable after matching; *p* = 0.915.
		Efanov et al., 2025. [17] Single-center PSM.	41 robotic vs. 82 open.	90-day mortality 5% vs. 5%; eight robotic cases converted; other short-term outcomes were not significantly different.	Median OS 44 vs. 29 months, *p* = 0.164.
		de Hondt et al., 2025. [37] Comparative systematic review/meta-analysis.	pCCA subgroup with 1185 pCCA patients (from 7 studies). MILS vs. Open.	Major morbidity 20.2% vs. 29.5% (OR 0.91, not significant); mortality 6.4% vs. 7.7% (OR 0.81, not significant); operation time 85 min longer; hospital stay 1.5 days shorter; blood loss not significantly different.	Recurrence was 62.8% vs. 63.1%, not significantly different. (These were whole-cohort results including iCCA.)
GBC, T1b/T2 and selected T3 without bile duct invasion	Extended cholecystectomy: gallbladder bed resection or Segment IVb/V resection + regional LND	Ielpo et al., 2024. [20] IRON international multicenter PSM.	123 robotic vs. 98 open 123 robotic vs. 155 laparoscopic	Robotic vs. open: Operation time 260 vs. 275 min; blood loss 183 vs. 320 mL; C-D ≥ III 7% vs. 12%; R0 89% vs. 84%; nodes 7 vs. 5. Robotic vs. laparoscopic: nodes 7 vs. 4.	OS and DFS were similar across approaches.
		Sohn et al., 2024. [21] Multicenter PSM.	69 MIS vs. 308 open.	Operation time 240 vs. 190 min; C-D ≥ III 9% vs. 12%; R0 92% vs. 93%; nodes 7 vs. 9.	5-year OS 57% vs. 69%; DFS 59% vs. 63%, without significant differences.
		Zhang et al., 2026. [47] Meta-analysis of five PSM studies.	629 patients, 202 robotic vs. 427 open radical cholecystectomies.	Fewer major complications (OR 0.42), lower blood loss (MD −114.77 mL), shorter stay (MD −2.92 days); no significant differences in mortality, overall morbidity, operation time, transfusion, or readmission, LN harvest (MD 0.13), or R0 (OR 1.14) were observed.	No significant differences in 3- or 5-year OS, or 3- or 5-year DFS.
		Kato et al., 2023. [22] Japanese registry.	1609 pT2 GBC; 520 EHBDR vs. 1089 non-EHBDR.	LNM 38.2% vs. 20.7%; C-D ≥ III 32.4% vs. 11.7%.	DSS 76% vs. 79%, *p* = 0.410; T2N1 subgroup 5-year survival 64% vs. 54%, *p* = 0.017.
GBC with invasion to cystic duct margin or extrahepatic bile duct	Extended cholecystectomy + EHBDR/CBDR + biliary reconstruction	Esposito et al., 2026. [49] International multicenter robotic PSM.	138 robotic GBC resections, 21 with CBDR. Robotic resections with vs. without CBDR	No significant differences in blood loss, conversion, bile leak, C-D ≥ III, LOS, LN yield, or 90-day mortality; LN yield 8.5 vs. 7.	OS and DFS were comparable after median follow-up of 17 months.
GBC with bile duct involvement or selected locally advanced disease	Major liver resection ± bile duct, vascular, or multivisceral resection	Vanterpool et al., 2026. [57] Single-center retrospective robotic series	38 robotic cases, including 9 CBD resections with Roux-en-Y hepaticojejunostomy.	No conversion; operation time 470 vs. 219 min with vs. without CBDR; median LN yield 6; R0 95%; vascular resection 33% vs. 3%.	Long-term conclusions were limited by sample size and follow-up.
dCCA	Pancreaticoduodenectomy + regional LND	Uijterwijk et al., 2023. [23] International PSM.	97 MIPD vs. 381 OPD.	Operation time 453 vs. 336 min; blood loss 300 vs. 420 mL; C-D ≥ IIIb 23% vs. 20%; nodes 14 vs. 14.	5-year OS 35% vs. 24%, *p* = 0.224; 5-year DFS 25% vs. 21%, *p* = 0.344.
		Xu et al., 2022. [24] Multicenter PSM.	217 RPD vs. 228 OPD; after PSM 180 vs. 180.	Blood loss 150 vs. 250 mL, *p* < 0.001; LOS 12 vs. 15 days, *p* < 0.001; no differences in LN harvest, R0, major morbidity, or 90-day mortality.	OS and RFS were similar.
		Gao et al., 2025. [25] PSM after learning curve.	430 total; 184 LPD vs. 184 OPD.	Blood loss 160 vs. 250 mL, *p* < 0.001; LOS 13 vs. 16 days, *p* < 0.001; no differences in R0, LN harvest, severe complications, 30-day mortality, or textbook outcome.	Long-term prognosis did not differ.
		Bakht et al., 2026. [26] Systematic review/meta-analysis.	Seven retrospective cohorts, 1803 patients.	MIPD reduced blood loss (WMD −100.86 mL) and LOS (WMD −2.46 days), but increased operation time (WMD 52.60 min); major complications OR 0.97 and mortality OR 0.46, neither significant.	OS HR 0.90 and DFS HR 0.98, neither significantly different.

Table 1 Footnote. BTC, biliary tract cancer; iCCA, intrahepatic cholangiocarcinoma; pCCA, perihilar cholangiocarcinoma; GBC, gallbladder cancer; dCCA, distal cholangiocarcinoma; MIS, minimally invasive surgery; MILR, minimally invasive liver resection; LLR, laparoscopic liver resection; OLR, open liver resection; LS, laparoscopic surgery; RS, robotic surgery; OS, overall survival; DFS, disease-free survival; RFS, recurrence-free survival; PD, pancreaticoduodenectomy; MIPD, minimally invasive pancreaticoduodenectomy; LPD, laparoscopic pancreaticoduodenectomy; RPD, robotic pancreaticoduodenectomy; OPD, open pancreaticoduodenectomy; PSM, propensity score matching; LND, lymph node dissection; LN, lymph node; LNM, lymph node metastasis; EHBDR, extrahepatic bile duct resection; CBDR, common bile duct resection; C-D, Clavien–Dindo classification; LOS, length of stay; WMD, weighted mean difference; HR, hazard ratio; OR, odds ratio; RR, risk ratio or relative risk according to context.

## 7. Conclusions

MIS for BTC should be evaluated according to operative task-load rather than disease subtype alone. The strongest current evidence supports selected use for peripheral iCCA and standard extended cholecystectomy for selected GBC. The evidence supporting MIPD for selected dCCA is also increasing. Central iCCA, pCCA, bile duct-involved GBC, and locally advanced GBC remain high-complexity fields requiring cautious application in expert centers. Robotics may facilitate difficult dissection and reconstruction, but a definitive oncologic advantage has not been demonstrated. Standardized task-based reporting and prospective multicenter data are required before broader adoption can be recommended.

## Data Availability

Data sharing is not applicable to this article because no new data were created or analyzed in this study. All data discussed in this review were derived from previously published studies.
